# The relations between dietary antioxidant vitamins intake and oxidative stress in follicular fluid and ART outcomes

**Published:** 2015-09

**Authors:** Ashraf Kazemi, Fatemeh Ramezanzadeh, Mohammad Hosein Nasr-Esfahani

**Affiliations:** 1 *Reproductive Health Department, Nursing and Midwifery Care Research Centere, School of Nursing and Midwifery, Isfahan University of Medical Sciences, Isfahan, Iran.*; 2 *Vali-e-Asr Reproductive Health Research Center, Imam Khomeini Hospital Complex, Tehran University of Medical Sciences, Tehran, Iran.*; 3 *Reproductive Biomedicine Center, Royan Institute for Animal Biotechnology, ACECR, Department of Reproduction and Development, Isfahan, Iran.*

**Keywords:** *Oxidative stress*, *Vitamin A*, *Vitamin C*, *Vitamin E*, *Assisted reproduction*, *Fertilization*, *Embryo quality*

## Abstract

**Background:**

Oxidative stress (OS) in the follicular environment may affect on oocyte competence and antioxidant vitamins may modify its effects.

**Objective:**

This study was conducted to examine the effect of dietary intake of vitamin A, C and E on OS in follicular environment and assisted reproduction technology (ART) outcomes.

**Materials and Methods:**

In this obsevationalprospective study, the intake levels of vitamin A, C, and E were matured by validated food frequency questionnaire and Malondialdehyde and the total antioxidant capacity (TAC) levels of follicular fluid (FF) in 219 women undergoing ART were assessed. The number of retrieved oocytes, percentages of metaphase II MII) stage oocytes, fertilization rate, and embryo quality were also determined.

**Results:**

No significant association was found between vitamins intake levels and OS biomarkers, but the mean of TAC level in FF among women who received vitamin C greater than 75 mg/d was higher than women with lower intakes (p<0.05). The ART parameters were not related to the vitamin E intake level, but the normal cleaved embryo rate was positively related to vitamin A (p<0.05) and vitamin C (p=0.02) intake levels. Also, the percentage of MII oocytes (p=0.02) and the fertilization rate (p<0.05) were related to the vitamin C intake level. The relation between the TAC level in FF and ART outcomes were not significant.

**Conclusion:**

Current results indicated that high dietary intake of vitamin C would be followed by increasing the TAC level in FF and improving the oocyte competence, but this effect of vitamin C is not dependent of increasing of antioxidant defense in follicular environment.

## Introduction

In vivo, oocyte maturation is influenced by the interplay of the biochemical state of the oocyte and its surrounding microenvironment (1). In an optimal condition the follicle constitutes a complex and regulates microenvironment in which the oocyte acquires developmental competence at ovulation (2, 3). This environment contains large numbers of macrophages, neutrophils, and metabolically active granulosa cells, which produce reactive oxygen species (4). These oxygen-derived molecules are intermediary products of physiologic cellular metabolism (5). Under normal conditions, these molecules are neutralized by a defense system consisting of enzymatic and non-enzymatic antioxidants (6). An overproduction of the reactive oxygen species and/or a deficit or depletion of the scavenging capacity of antioxidant defense systems leads to oxidative stress (OS) and oxidative damage of key intracellular components (7).

Some studies have suggested that OS in antral follicle may induce follicular atresia (8, 9) and influences oocyte competence (10-13). Therefore, the efforts aimed at reducing OS in a follicular environment can be considered. Some evidence suggests that dietary intervention could be an effective strategy to reduce systemic OS and its cellular damages (14, 15). Antioxidants are important in maintaining the oxidant–antioxidant balance in blood and tissues. The antioxidants vitamins, such as vitamins A, C, and E are important in restoring or maintaining the redox balance in blood and tissues (16).

In addition, for the embryo to acquire developmental potential, it may be important that antioxidants are stored in the oocyte during its growth and maturation phases. Tola *et al*. suggested that high intracellular antioxidant activity in granulosa cells had a positive effect on oocyte competence for fertilization (17). As a result, dietary antioxidant availability due to a rich antioxidant dietary intake could have an impact on the level of the OS in follicular environment.

Much of the interest in improving the quality of in vivo and in-vitro-matured human oocytes has focused on the effects of antioxidant supplementation (18). While, the dietary sources of vitamin intake, in addition to supplying vitamin, could have other metabolic effects (19). The effects of dietary antioxidant-rich intake on OS in the follicular environment have scarcely been investigated; besides diets rich with antioxidants deserve consideration in the context of the OS in follicular environment. The previous findings on the effect of ascorbic acid upon reproduction were contradictory. While, some studies in animal models showed the positive effects of ascorbic acid on the oocyte competence (20, 21), the results of other studies have not confirmed these findings (22). Also, some documents have shown the positive effect of using supplemental vitamin A on ART outcomes (23, 24). But there is no evidence to show that the positive effects of this vitamin on reproduction looking for antioxidant characteristics of vitamin A.

Therefore, the relationship between the dietary antioxidant vitamins intake level and lipid peroxidation product in FF, in addition, the relationship between the dietary antioxidant vitamin intake and assisted reproductive parameters were evaluated.

## Materials and methods

For this observational prospective study, 219 non-donor in vitro fertilization/intra-cytoplasmic sperm insemination (IVF/ICSI) cycles were randomly conducted from July 2010 to April 2011 at Isfahan Fertility and Infertility centre. The study was approved by the Institutional Review Board and the Ethics Committee of Isfahn University of Medical Sciences. The exclusion criteria were male factor infertility, according to world health organization (WHO) criteria (25), known systemic disease and considerable change in dietary pattern during assisted reproduction cycle. All eligible women gave written informed consent.

**Assessments**

At the start of ART cycle a validated semi-quantitative food frequency questionnaire (FFQ) including 168 food items (26) was used to measure calorie intake and vitamin intake, including vitamin A, C and E over the previous three months.

For all the main food items in the FFQ, the frequency per day was multiplied by the amount consumed, depending on the portion size, to compute the total amount consumed per day. The United States department of agriculture food composition table was used for most items. Depending on the appropriate vitamin intake, the subjects were divided into two groups for each vitamin.

For lifestyle variables, the participants were asked about smoking status (passive and active smoking) and about vitamin supplement (use Yes/No).

**Assisted reproduction**

Ovarian hyperstimulation using long protocol, involving gonadotropin releasing hormone agonist (Suprefact, Hoechst, Germany) and recombinant follicle stimulating hormone (Gonal F, Serono, Rockland, MA, USA, Follistim, Organon, Roseland, NJ) and/or human menopausal gonadotropin (Menogon, Ferring, Suffern, NY) administration were done and follicular maturation was monitored by transvaginal ultrasound examination.

Ovulation was triggered with 10,000 IU human chorionic gonadotropin (Pregnyl, Organon), when dominant follicles reach a follicular size of around 18mm. 36 hours later, the oocytes were retrieved transvaginally and used for IVF or ICSI procedure. After retrieving oocytes, 2 ml follicular fluid from one to 5 follicles (with ≤16 mm diameter) was pooled and centrifuged at 300 rpm for 17 miN. of the supernatant was stored at −70^°^C. Maximum storage time was two weeks. Bloody samples and samples without oocyte were discarded.

Despite exclusion criteria of male factors infertility, as a routine protocol of Isfahan Fertility and Infertility center, some of oocytes were inseminated through ICSI procedure.

Oocytes were considered fertilized when two pronuclei was observed 17-19h post sperm insemination. According to Veeck's criteria high-quality embryos at 3 days after oocyte retrieval were defined as grades one and two (27). The percentage of embryo with good quality was calculated by dividing the number of high-quality embryos by the number of total embryos. Embryos with more than 5 cells on 3 days post IVF/ICSI were considered as normal cleaved embryos and the normal cleaved embryos rate was calculated by dividing the number of the normal cleaved embryos by the number of total embryos. In ICSI cycles, the percentages of the MII stage were calculated by dividing the number of oocytes with first polar body divided by the number of assessed oocytes. The data about the number of used gonadotropin were recorded based on the patients' medical file.

**Laboratory analyses**

The levels of Malondialdehyde (MDA) and TAC in FF were considered as OS markers and have been assessed in Isfahan Cardiovascular Research Institute, Iran. Aliquots of the FF were thawed at room temperature and follicular fluid lipid peroxidation was assessed by MDA assay according to Oral et al (28). The TAC was assessed by enhanced chemiluminescence assay as described previously (29). All the samples were protected from direct sunlight.

**Statistical analysis**

Statistical analysis was performed using the Statistical Package for the Social Sciences, version 13.00, SPSS Inc, Chicago, Illinois, USA (SPSS software). The normality of the vitamin intake level distributions was skewed. Therefore, normality of data was improved by using log transformation. Descriptive analyses were reported using mean and standard deviation. The data were analyzed using linear regression (adjusted for age, body mass index (BMI), smoking status, calorie intake and etiology of infertility). Also, the daily vitamin intakes (vitamin A, C, and E) were divided into two groups according to the recommended dietary allowances and adequate intakes for adult females (USDA, Release 11, 1994) and the ART outcomes have been compared between the two groups using t-test. The P value of less than 0.05 was considered to be significant.

## Results

In total, 219 subjects with mean age 31.54 ± 6.20 years participated in the study. The dietary vitamin intake levels in subjects and assisted reproduction outcomes were presented in table I.

Associations between the levels of vitamin intake and OS biomarkers in FF and ART parameters adjusted for age, physical activity, BMI, passive smoking status, etiology of infertility, and calorie intake are presented in table II.

The association between the vitamin intake levels and the MDA and TAC levels in FF were not significant. 

Among the ART parameters, independent of the MDA and TAC levels in FF, the normal cleaved embryo rate was related to the vitamin A intake (p<0.05); and the MII stage oocyte rate (p=0.02), fertilization rate (P<0.05) and percentage of good cleaved embryo (p=0.02) were related to the vitamin C intake level positively.

In table III, the comparison of the OS biomarkers level in FF and ART parameters of 219 participants by the vitamins (vitamin A, C, and E) intake groups are shown. The mean of TAC level in FF among women who received vitamin C greater than 75 mg/d was higher than women with lower intakes.

The normal cleaved embryo rate was significantly higher in women with a vitamin A intake over 700 µg/d than lower intakes. The MII stage oocyte rate, fertilization rate and percentage of non-fragmented embryo were significantly higher in women with a vitamin C intake over than 75 mg/d than lower intakes. 

**Table I.** Personal details, the level of the vitamin intake, and ART parameters in subjects

**Variables **	**Mean±SD or %**
Age (year)	31.54 ±5.45
BMI (kg/m2)	26.60 ±4.33
Duration of infertility (year)	7.42±5.14
**Etiology of infertility (%)**	
Anovulation	40.64
Pelvic factor	46. 58
Hypothalamic amenorrhea	5.48
Unexplained infertility	7.30
**Vitamins intake level**	
Vitamin A (µg/d)	1445.90 ±1684.50
Vitamin C (mg/d)	81.38±79.86
Vitamin E (mg/d)	22.84±18.45
**Oxidative stress markers**	
MDA (µ mol/lit)	0.98±0.29
TAC (molar Trolox equivalents)	1987.73±354.08
**ART parameters**	
Used gonadotropin (n)	40.54±1.72
Retrieved oocytes (n)	11.95±9.11
Fertilization rate (%)	62.60 ±27.10
Non-fragmented embryo rate (%)	44.81±36.21
Number of blastomers	6.13±2.44
Normal cleavage rate (%)	71.50±36.60
Good quality embryo rate (%)	47.14±34.01

BMI: Body mass index, MDA: Malondialdehyde, TAC: Total antioxidant capacity, ART: Assisted reproductive technology.

**Table II:** The correlation between dietary vitamin intake levels, oxidative stress markers and assisted reproduction parameters

	**Number ** **of oocytes**			**MII ** **Rate**			**Fertilization ** **rate**			**Normal cleaved embryo rate**			**Good embryo** ** rate**			**Non- fragmented embryo rate**		
	**Beta**	**95% CI**		**Beta**	**95% CI**		**Beta**	**95% CI**		**Beta**	**95% CI**		**Beta**	**95% CI**		**Beta**	**95% CI**	
**Vitamin A (µg/d)**	-0.11	-5.7	0.9	0.04	-8.5	9.5	-0.1	-0.2	0.2	0.2	0.1	12	0.07	-2.2	0.03	0.79	-9.2	16.1
**Vitamin C (mg/d)**	0.12	-0.6	5.3	0.2**	0.1	10	0.16*	0.01	0.3	0.2**	0.1	13.1	0.09	-5.4	18.9	0.02	-2.4	21.1
**Vitamin E (mg/d)**	-0.03	-5.4	3.6	-0.1	-17	5.4	0.02	-0.2	0.3	-0.1	-13	5.9	-0.07	-20	10.3	0.01	-10	10.2
**MDA**	-0.8	-0.7	2.2	-0.1	-1	10.7	-0.1	-0.4	0.1	-0.1	-33	0.14	-0.08	-31	9.2	-0.06	-18	13.8
**TAC**	-0.3	-0.01	0.003	0.06	-0.01	0.01	-0.1	-0.09	0.01	-0.02	-0.02	0.01	0.04	-.01	0.02	0.08	-0.01	0.02

CI: Confidence Interval, Beta: Standardized coefficient, MDA: Malondialdehyde, TAC: Total antioxidant capacity, MII: metaphase II.

* p<0.05;

** p=.02 (Multiple regression analysis)

**Table III:** Comparison of oxidative stress markers and ART parameters by dietary vitamin intake categorisations

	**Vitamin A intake levels** **(µg/d)**		**p-value**	**Vitamin C intake levels** **(mg/day)**		**p-value**	**Vitamin E intake levels** **(mg/day)**		**p-value**
	**≤700**	**>700**		**≤75**	**>75**		**≤15**	**>15**	
**Number**	147	72		149	70		163	56	
**MDA**	0.99± 0.27	0.98± .31	0.55	0.97± 0.96	1.03± 0.82	0.17	0.99± 0.27	0.96± 0.33	0.21
**TAC**	1999.00± 345	1962± 375	0.21	1957.01± 380.02	2050.00± 283.11	0.04	1997.01± 343.11	1957± 387	0.15
**No. of oocytes**	12.11± 8.72	11.7± 9.60	0.19	11.81±9.03	12.90± 9.28	0.38	12.10± 9.41	11.6± 8.40	0.35
**MII Rate**	82.00± 23.92	83.5± 15.51	0.20	79.01 ± 25.22	87.13± 14.81	0.008	83.24± 22.22	80.4± 23.70	0.20
**Fertilization rate**	65.62± 64.11	65.5± 63.41	0.28	60.31± 28.71	71.72± 22.41	0.04	64.9 ± 26.60	67.4± 29.30	0.09
**Normal cleavage embryo rate**	71.62± 36.21	81.9± 27.41	0.02	73.00± 35.31	80.5± 34.41	0.36	75.50± 34.23	73.5± 33.70	0.17
**Good embryo rate**	48.32± 36.90	52.11± 38.70	0.12	46.20± 36.51	55.80±34.40	0.18	49.13± 38.41	50.8± 35.10	0.36
**Non-fragmented embryo rate**	45.00± 35.32	50.2± 37.51	0.10	43.11± 35.42	56.21± 35.81	0.02	45.60± 35.81	49.92± 37.31	0.07

All data were presented as Mean±SD. ANOVA test.

ART: Assisted reproductive technology, MDA: Malondialdehyde, TAC: Total antioxidant capacity, MII: metaphase II.

## Discussion

To determine the effect of dietary antioxidant vitamin intake on assisted reproduction treatment by influencing on the OS in follicular environment, two objectives were followed, including: the relations between the dietary vitamins A, C, and E intake levels and the measured the OS markers in FF and the relation between the dietary vitamin A, C and E intake and assisted reproductive parameters. Initially, the result of this study revealed that a relationship between intake of vitamins A, C and E intake and MDA and TAC in FF were not linear. However, in women with higher intakes of vitamin C level than the recommended levels (>75 mg/day), the TAC level was significantly higher. Although, the effect of vitamins A, vitamin C and, vitamin E on maintaining the oxidant antioxidant balance in blosod and tissues was demonstrated previously (16), these findings suggested that only dietary vitamin C intake may have a positive effect on antioxidant defense in follicular microenvironment. 

Other findings revealed that the oocyte competence such as the oocyte maturation and fertilization were improved by more vitamin C intake, independent of the OS markers levels in FF. Also, higher dietary intake of this vitamin was associated with decreasing embryo fragmentation. The present observations were consistent with the previous studies showing protective effects of vitamin C supplementation on MII mouse oocyte spindle structure and chromosomal alignment against an oxidative damage of hydrogen peroxide (30) and blocking the DNA damage in treating oocytes by ROS (31). Also, increased levels of glutathione peroxidase and vitamin C concentration, and reduced lipid peroxidation have been reported in FF of women who undergoing IVF program with multivitamin and mineral supplementation (18).

In some other reports, the beneficial effect of vitamin C on oocyte maturation was not supported. According to Dalvit et al, the use of the antioxidants vitamin E and C during in vitro maturation in bovine did not affect the percentage of nuclear maturation and fertilization (22). However, this study suggested that the assisted reproduction outcomes were not related to dietary intake level of vitamin E.

Therefore, the beneficial effect of vitamin C on ART outcome might not depend on the antioxidant effect of this vitamin. The effects of vitamin C on ART outcomes might be explained by anti-apoptotic effect of vitamin C on oocyte–granulosa cell complexes (32) and its role in regeneration of vitamin E has been demonstrated previously (33).

Also, this study revealed that although the association between the TAC level in FF and the vitamins A intake level was not significant, the percentage of normal cleaved embryo was positively related to vitamin A intake level. Also, these evidences suggested that the oocyte’s developmental competence could be enhanced by vitamin A intake during intra follicular growth.

In consist with these findings, some researchers have suggested that antioxidants, including vitamins despite their antioxidant capacity might exert their effect on oocyte competence through other routes (18, 33). Combelles et al. suggested that in-vivo supplementation with vitamins that possess antioxidant activity did not necessarily improve reproduction (34).

The cytoplasmic retinol-binding proteins existed in human ovaries (35) and high concentration of vitamin A in human FF (36) suggested that vitamin A had an important role in reproductive function.

## Conclusion

This paper described the impact of the dietary intake of vitamins on the oxidative protection in FF and on the oocytes-embryos parameters. The vitamins C and A might be useful to generate better eggs and embryos.
